# Estimating disease-free survival of thyroid cancer based on novel cuprotosis-related gene model

**DOI:** 10.3389/fendo.2023.1209172

**Published:** 2023-09-08

**Authors:** Rui Du, Jingting Li, Fang Li, Lusi Mi, Gianlorenzo Dionigi, Hui Sun, Nan Liang

**Affiliations:** ^1^ Division of Thyroid Surgery, The China-Japan Union Hospital of Jilin University, Jilin Provincial Key Laboratory of Surgical Translational Medicine, Jilin Provincial Precision Medicine Laboratory of Molecular Biology and Translational Medicine on Differentiated Thyroid Carcinoma, Changchun, China; ^2^ Department of Pathophysiology and Transplantation, Division of Surgery, Istituto Auxologico Italiano IRCCS (Istituto di Ricovero e Cura a Carattere Scientifco), University of Milan, Milan, Italy

**Keywords:** Cuprotosis, thyroid cancer, Prognosis, immune cell infiltration, survival, genes, model

## Abstract

**Background:**

Cuprotosis is a newly discovered form of cell death that differs from other types of cell death. The aim of this study was to investigate the functional role and a possible prognostic model for thyroid cancer.

**Methods:**

TCGA and GEO were used to investigate the differential expression of CRGs in THCA. KEGG and GO enrichment analyses were applied to investigate the possible molecular functions. The features of CRGs were selected by LASSO regression. 20 pairs of samples were randomly collected from the hospital to compare expression between tumor and normal.

**Results:**

Among the 19 CRGs related to thyroid cancer recurrence, 16 genes were differentially expressed in thyroid cancer. KEGG analysis showed that the 19 CRGs were mainly enriched in cell death, cell cycle and ribosomal pathways. K-M survival analysis and subsequent multiple logistic regression revealed that the expression of BUB1 and GINS2 were potential risk factors for disease-free survival (DFS) of thyroid cancer. In addition, further LASSO-regression selected the following three DFS-related CRGs: FDX1, BUB1 and RPL3. A novel prognostic prediction model was constructed by nomogram, and the prediction probability for 1-, 3- and 5-year survival approached the actual time. As for the possible mechanisms, FDX1, BUB1 and RPL3 were associated with immune infiltration. The cell model experiment illustrated that the ATM signaling pathway might be involved in thyroid cancer cell death.

**Conclusion:**

Three CRG models (FDX1, BUB1, RPL3) could better predict the prognosis of thyroid cancer. Immune cell infiltration and the ATM pathway were the possible mechanisms.

## 1 Introduction

The incidence of thyroid cancer has increased in recent years (1). The mortality rate of thyroid cancer is low, but the recurrence rate is high, which is a major challenge for patients and surgeons. Moreover, the specific molecular mechanisms of thyroid cancer (THCA) are still unclear. New efficient prognostic model using biomarker panels are required.

Cuprotosis is a newly discovered cell death that depends on the accumulation of copper ions and leads to programmed cell death (**Table S1**). Copper and other trace metals are essential for life. Excessive accumulation of metal ions is life-threatening. However, abnormal concentrations of copper and zinc ions have been found in the serum of thyroid cancer patients (2, 3), so we hypothesized that the disturbance of copper ion metabolism in thyroid cancer patients may be one of the risk factors for tumor development. Furthermore, there is more than one type of programmed cell death, including necrosis, apoptosis, autophagy and iron death, and all types of cell death often interact directly with each other. The potential relationships and interactions with other types of cell death in thyroid cancer may provide us with some new ideas.

The aim of this study was first to investigate the expression of Cuprotosis-related Genes (CRGs) in thyroid cancer and then to build a prognostic model to predict the prognosis of thyroid cancer. Finally, the possible molecular mechanism in the cell model is also investigated.

## 2 Materials and methods

### 2.1 Data collection

RNA sequencing expression profiles and corresponding clinical information for thyroid cancer were downloaded from TCGA (**Table S2**). Statistical analyses were performed using R software v4.0.3. *P*<0.05 was considered statistically significant (4). CRGs were downloaded from Tsvetkov’s research (5). The GSE54958 dataset is from the GEO database (6, 7).

### 2.2 Functional enrichment analysis

We used the GO annotation of genes in the R software as a background to assign genes to the background set. The R software package cluster Profiler was used for enrichment analysis to obtain the gene set enrichment results. For gene set enrichment function analysis by using gene annotation of the latest KEGG Pathway. The minimum gene set was set to 5, the maximum gene set was set to 5000, and the *P* < 0.05 and FDR < 0.25 were considered statistically significant.

### 2.3 Analysis of immune infiltration

We used the immune genes module of the TIMER2 web server to investigate the association between the expression of BUB1, FDX1 and RPL3 and immune infiltration in thyroid tumors. Immune cells B cells, CD8+ T cells, CD4+ T cells, macrophages, neutrophils and dendritic cells were selected. P-values and partial correlation values (cor) were determined using the purity-adjusted Spearman rank correlation test. We used the TIDE platform (http://tide.dfci.harvard.edu) to evaluate the rank of the gene set in another database.

### 2.4 Protein-protein interaction network analysis

STRING was used to perform the PPI network analysis for different genes. The PPI network was visualized using Cytoscape software (version 3.9.0).

### 2.5 Immunohistochemistry

The Human Protein Atlas database (www.proteinatlas.org) was used to obtain the immunohistochemistry of FDX1, RPL3, AURKA, CDKN2A, DERL2, NOM1, PDHA1, GINS2, GPI, RPL10, PSMA6, RAD50, RAD9A, QARS1, SLC26A6, ZNHIT2 and TTK in tumor and normal tissues. Only BUB1 and TUT1 are not found in the Atlas database.

### 2.6 Survival analysis

The KM survival analysis with the log-rank test was also used to compare the survival differences between the two groups mentioned above and to plot the DFS curves. Cox regression analysis was performed to find out the risk factors that may predict DFS (8, 9).. For the Kaplan-Meier curves, p-values and hazard ratio (HR) with 95% confidence interval (CI) were obtained by log-rank tests and univariate Cox proportional hazards regression. All the above analysis methods and R packages were performed using R software version v4.0.3.

### 2.7 Prognosis model

The Least Absolute Shrinkage and Selection Operator regression algorithm (LASSO) was used for feature selection, 10-fold cross-validation was performed, and the R package GLMNET was used for analysis (8, 10–12). Further, there are 360 samples were used for the subsequent analysis. The log-rank test was used to compare survival differences between these groups. The time analysis ROC (v 0.4) was used to compare the predictive accuracy of the BUB1, FDX1 and RPL3 genes with the risk score. Multivariate Cox regression analysis was used to build a prediction model, and the R package survival was used for the analysis.

Univariate and multivariate Cox regression analyses were performed to determine the correct terms to construct the nomogram. The R package forest plot was used to plot the P-value, HR and 95% CI for each variable. Based on the results of the multivariate Cox proportional hazards analysis, a nomogram was developed to predict X-year overall recurrence (12). The nomogram provided a graphical representation of the factors that can be used to calculate the risk of recurrence for an individual patient based on the scores associated with each risk factor using the R package “RMS”.

### 2.8 Sample collection

20 pairs of samples were collected from the Department of Thyroid Surgery, China-Japan Union Hospital, Jilin University (**Table S3**) which were selected from April 2022 to December 2022. Patients with PTC who had undergone surgery and had a pathological diagnosis were included in the study. The inclusion criteria were as follows: (1) first thyroid surgery was performed in our department; (2) postoperative paraffin pathology was diagnosed as PTC; (3) BMI:18.5-23.9. The exclusion criteria were as follows: (1) patients with other malignancies; (2) lack of required relevant information.

The study was conducted in accordance with the Declaration of Helsinki and approved by the China-Japan Union Hospital Institutional Review Board (No.20220804014). Informed consent was obtained from all patients. The study was conducted in accordance with the Declaration of Helsinki. Both tumor samples and para-carcinoma normal tissue samples were collected 30 minutes after surgery, immediately placed in sterilized vials, frozen in liquid nitrogen and stored at -80 °C.

### 2.9 Quantitative real-time PCR

Total RNA was extracted from tissues via TRIzol (TAKARA, Beijing, China), followed by reverse transcription into cDNA. PCR was carried out using the TB Green R Premix Ex TaqTM II kit (TAKARA, Beijing, China). The reaction procedures were as follows: 32 cycles at 94◦C lasting 15 s, 60◦C lasting 10 s, and 72°C lasting 20 s. GAPDH were served as the loading control.

### 2.10 Cell culture

Human thyroid cancer cell line (TPC-1) was cultured in RPMI-1640, comprising 10% fetal bovine serum (FBS) at 37 °C and 5% CO2.

### 2.11 Cell viability assay

Cells (3000 to 4000/well) were seeded in 96-well plates. After a 24 h culture, cells were treated with different doses of I 131 for 24 h. The Cell Counting Kit-8 assay was then carried out to assess the cell viability.

### 2.12 The western blot analyses

Cells were lysed using Total Protein Extraction Kit. Protein concentration was quantified using a BCA Protein Assay kit. Equal amounts of protein were subjected to sodium dodecyl sulfate polyacrylamide gel electrophoresis before electro‐transfer to a polyvinylidene difluoride membrane. After blocking with 5% skimmed milk, membranes were probed overnight at 4°C with specific primary antibodies, before incubation with corresponding secondary antibodies, and protein was detected with an enhanced chemiluminescence method. The specific proteins included P- ATM, ATM, MAPLC3, P62, PI3KC3 and γ-H2AX. GAPDH was used as a control.

### 2.13 Statistical analysis

Continuous variables were described as mean ± standard deviation. Categorical variables were described as frequencies and proportions. Statistical differences between 2 populations were calculated using t-tests (2-sided) including multiple t-tests, unpaired t-tests or paired t-tests. Categorical data were analyzed using the chi-square test or the chi-square test with continuous correction. P<0.05 was considered statistically significant.

## 3 Results

### 3.1 Differential expression of CRGs in THCA

**Figure 1:**
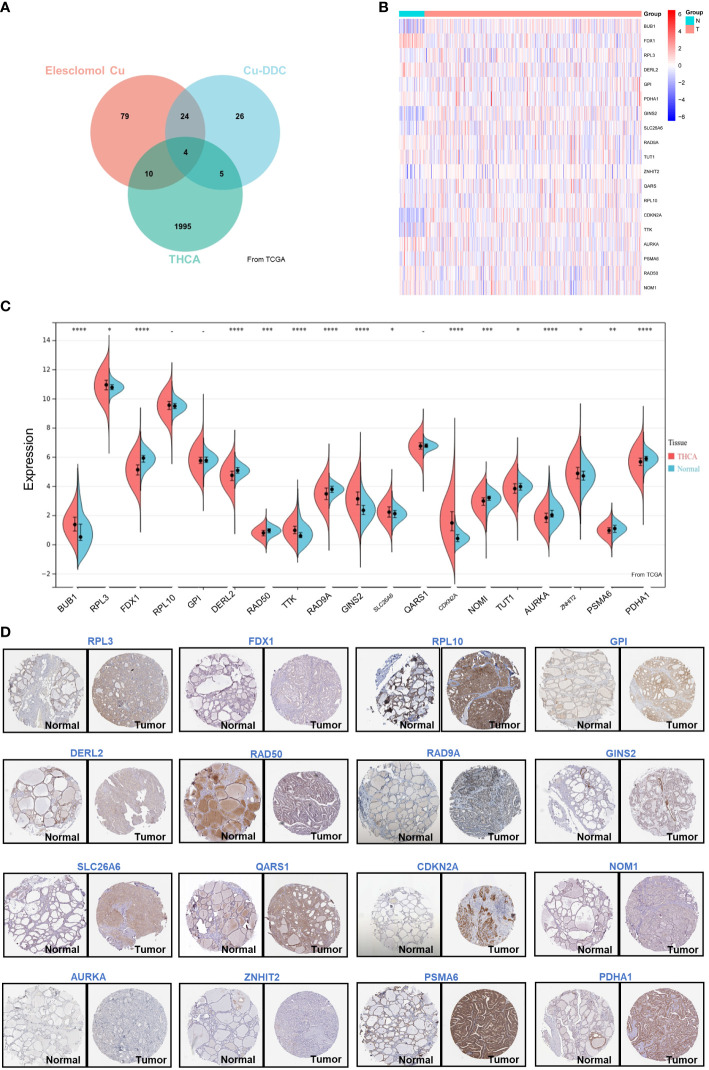
Differential Expression of CRGs in THCA. **(A) **Venn plot of the prognosis-related genes of THCA and CRGs; **(B)** Heatmap of the CRGs associated with DFS; **(C)** The expression of 19 genes in thyroid cancer and normal tissues; **(D)** The expression of RPL3, FDX1, RPL10, GPI, DERL2, RAD50, RAD9A, GINS2, SLC26A6, QARS1, CDKN2A, NOM1, AURKA, ZNHIT2, PSMA6, and PDHA1 showed by immunohistochemistry in thyroid cancer and normal tissues. *p < 0.05, **p < 0.01, ***p < 0.001, ****p < 0.0001.

To investigate the expression of CRGs in thyroid cancer, both the mRNA level and the protein level were analyzed. First, 148 previously reported significant CRGs were compiled from the literature(Elesclomol Cu and Cu-DDC) (5). We also analyzed the survival probability of thyroid cancer patients’ gene expression (2014) using the Cancer Genome Atlas (TCGA) database. As shown in the Venn diagram (**Figure 1A**), 19 CRGs overlapped with the recurrence-relevant genes in thyroid cancer. Therefore, we focused on these 19 CRGs associated with thyroid cancer recurrences in the following study. Based on the TCGA database, we first collected and compared the mRNA expression levels. From the heatmap (**Figure 1B**) and boxplot (**Figure 1C**), we found that of the 19 CRGs associated with thyroid cancer, 16 genes were differentially expressed in thyroid cancer. Compared with normal thyroid tissue, 7 CRGs were more highly expressed in thyroid cancer, namely BUB1 (*P*<0.001), RPL3 (*P*<0.001), TTK (*P*<0.001), GINS2 (*P*<0.001), SLC26A6 (*P*<0.001), CDKN2A (*P*<0.001) and ZNHIT2 (*P*<0.001). Meanwhile, 9 CRGs were less expressed, such as FDX1(*P*<0.001), DERL2(*P*<0.001), RAD50(*P*<0.001), RAD9A(*P*<0.001), NOM1(*P*<0.001), TUT1(*P*<0.001), AURKA(*P*<0.001), PSMA6(*P*<0.001) and PDHA1(*P*<0.001). In addition, we further compared and validated the protein level based on the human protein database ATLAS. As shown in **Figure 1D**, the majority of proteins were differentially expressed in thyroid cancer, which was consistent with the mRNA expression level. The above data suggest that the 19 CRGs may play an important role in thyroid cancer.

### 3.2 Functional enrichment and KEGG analysis of CRGs in THCA

**Figure 2:**
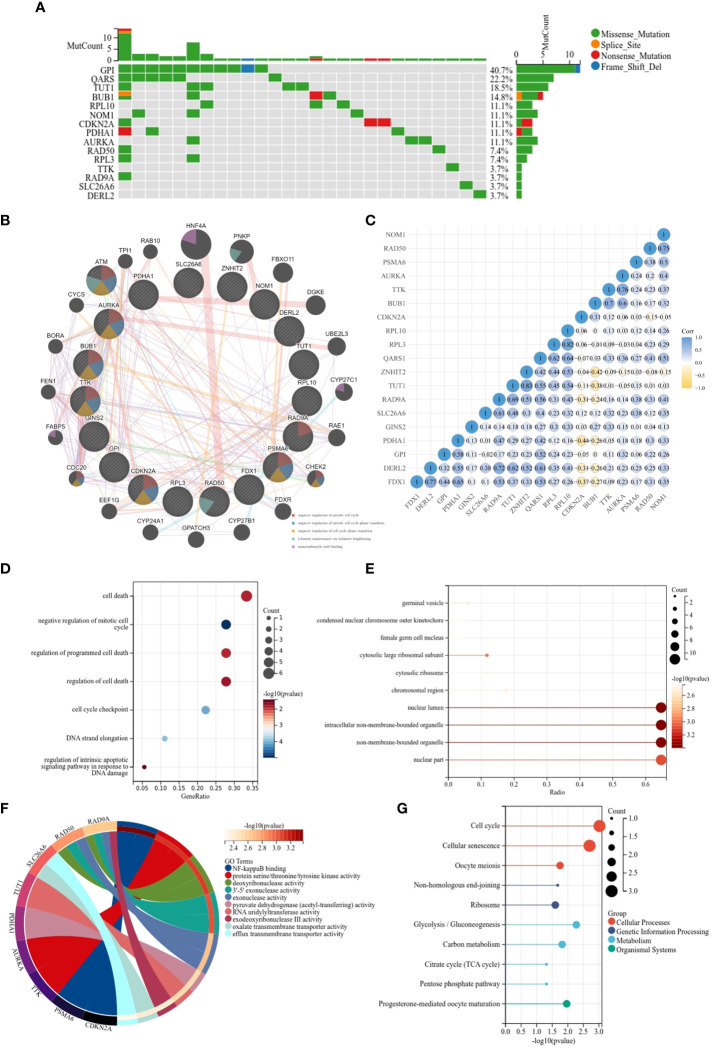
Functional enrichment and KEGG analysis of CRGs in THCA. **(A)** The mutation frequency and classification of 19 CRGs in THCA; **(B)** The PPI network provided interactive information among the 19 CRGs; **(C)** The interaction relationship among 19 CRGs; **(D)** KEGG pathway enrichment analysis**;(E–G)** MF, CC, BP- Gene ontology (GO) pathway enrichment analysis.

Analysis of the genetic variants revealed that a total of 15 genes were mutated, with the most common type of mutation being a missense mutation (**Figure 2A**). To investigate the correlation between the 19 genes, we created a PPI network using the STRING database and Cytoscape 3.9.0 software. The PPI network (**Figure 2B**) has 39 nodes and 277 edges. The interaction value > 0.15 was considered an interaction relationship with high reliability. The network showed that BUB1, RPL3 and FDX1 are the core genes. In addition, the correlation of the CRGs was examined using the ACLBI database (**Figure 2C**). To further investigate their potential biological functions, GO and KEGG analyses were performed within the 19 CRGs (**Figures 2D–G**). In the analysis of GO, the biological processes of the 19 CRGs were mainly cell death, regulation of programmed cell death, negative regulation of the mitotic cell cycle and regulation of the intrinsic apoptotic signaling pathway in response to DNA damage. However, according to KEGG analysis, they may also be involved in the cell cycle, cellular senescence, oocyte meiosis and glycolysis/gluconeogenesis. The above analysis suggests that these 19 CRGs may play an important role in the progression and development of THCA.

### 3.3 Predictive efficacy of CRGs for survival in THCA

**Figure 3:**
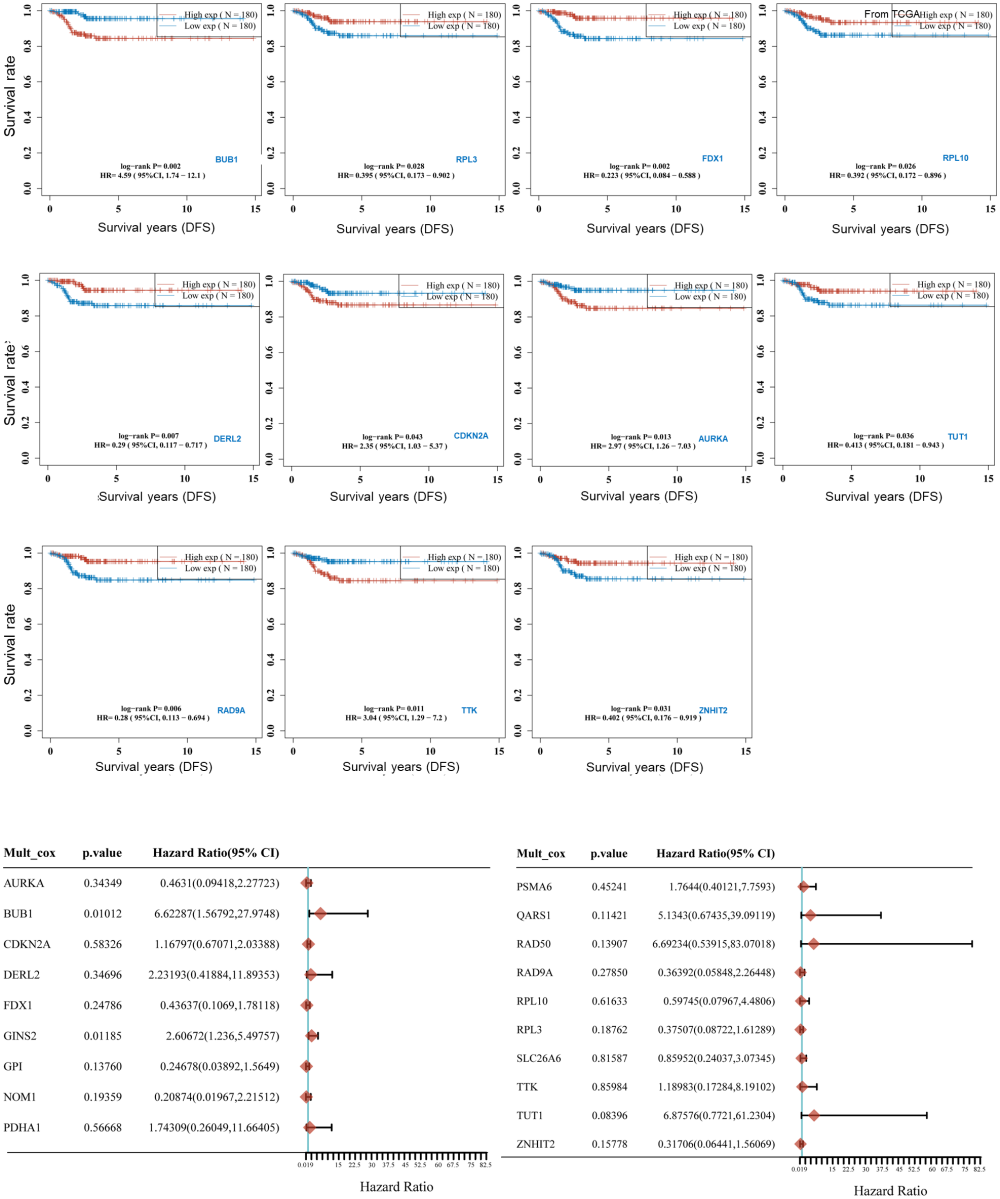
Predictive efficacy of CRGs for Survival in THCA. **(A)** Kaplan-Meier curves for high and low expressed groups in the two subgroups of the CRGs; **(B)** Multivariate Cox regression analyses established that CRGs expression exerted a critical influence on the Disease-free survival (DFS).

To uncover the relationship between prognosis and expression of CRGs and to find the best predictors for disease-free survival of thyroid cancer, K-M curve analysis and Cox regression were applied. As shown in **Figure 3A**, four CRGs correlated positively with disease-free survival of THCA as follows: BUB1 (HR=4.5867 (1.7368-12.1126), *P*=0.0021), TTK (HR=3.0436 (1.2868-7.1989), *P*=0.0113), CDKN2A (HR=2.3511 (1.0291-5.3712), *P*=0.0426), AURKA (HR=2.9729 (1.2569-7.0315), *P*=0.0131). Taking BUB1 as an example, patients with higher expression of BUB1 could have poorer survival. However, there were seven other CRGs that correlated negatively with DFS. These were RPL3, FDX1, RPL10, DERL2, RAD9A, TUT1 and ZNHIT2. Further multivariate regression analysis (**Figure 3B**) revealed that BUB1 and GINS2 were positively associated with the DFS of THCA. Of note, THCA with high BUB1 expression was 6.623 times more likely to have a worse prognosis than patients with low expression. This suggests that these CRGs are a potential risk factor for DFS in THCA.

### 3.4 Building and validating a prognostic model based on CRGs in THCA

**Figure 4:**
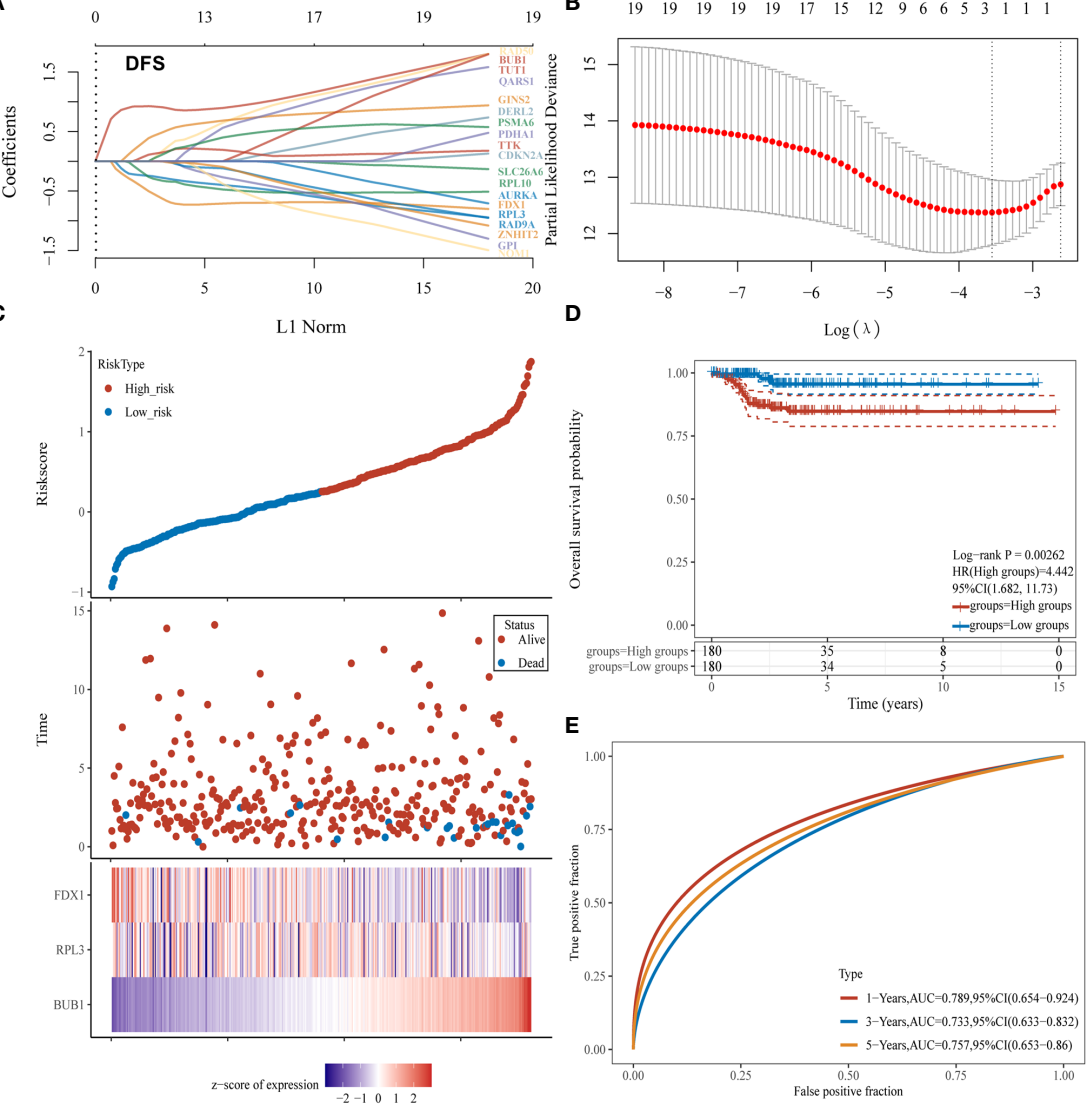
Establishment of a CRGs model for predicting the prognosis of thyroid cancer patients. **(A, B)** Fitting processes of LASSO Cox regression model of CRGs; **(C)** The ranking of the risk scores among all thyroid cancer samples; **(D)** Kaplan-Meier disease-free survival analysis for high (red) and low (blue) risk groups; **(E)** ROC for 1- (red), 2- (blue) and 3-year (yellow) survival time for high and low-risk patients.

To build a CRG model to predict the prognosis of thyroid cancer patients, LASSO Cox regression analysis and nomogram were applied. Three CRGs (FDX1, RPL3, BUB1) were selected to build the prognostic model (**Figure 4A**) As shown in **Figure 4B**, the model had the optimal performance and the least number of independent variables when log λ was set to 0.0287. The risk score was calculated as follows: Risk score = (0.7896) *BUB1+(-0.0151) * RPL3+(-0.1247) * FDX1. According to the median value of all risk scores, 360 thyroid cancer patients were divided into high and low-risk score groups. (**Figure 4C**). A heat map visualized the expression patterns of the three CRGs between high and low-risk groups (**Figure 4C**). As shown by the K-M curves, the risk in the high-risk group was 4.442 times higher than in the low-risk group (*P*= 0.00262; **Figure 4D**). To further validate the diagnostic efficacy of the prognostic model, we created a ROC analysis. The result confirmed that the CRG model could predict DFS probability for 1-year [AUC = 0.789, 95%CI (0.654-0.924)], 3-year [AUC = 0.733, 95%CI (0.633-0.832)] and 5-year survival [AUC = 0.757, 95%CI (0.653-0.860)] (**Figure 4E**). Overall, this Cuprotosis-related three-gene model could be a robust prognostic model for thyroid cancer.

**Figure 5:**
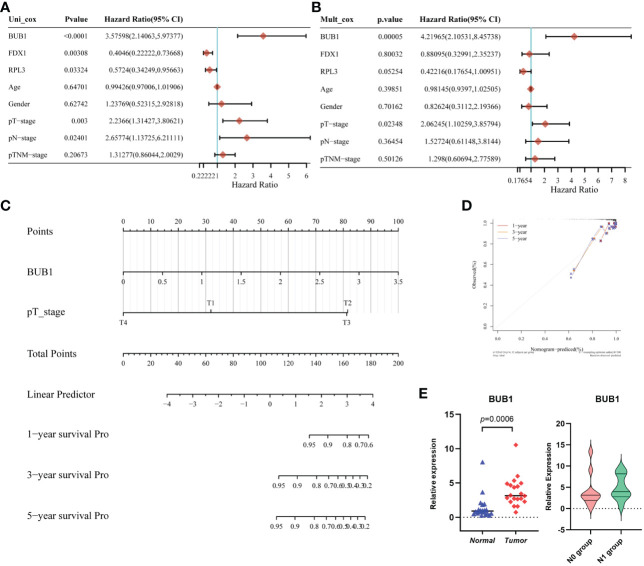
Establishment and validation of the Prognostic Model based on CRGs in THCA. **(A)** The univariate Cox regression of CRGs and clinical characteristics; **(B)** The multivariate Cox regression of CRGs and clinical characteristics;**(C)** the Nomogram of 1-year, 2-year, and 3-year DFS prediction of Thyroid cancer patients. C-index: 0.81(0.721-1) *P* < 0.001; **(D)** Calibration curve for the disease-free survival nomogram model in the discovery group; **(E)** The validation of relative expression of BUB1 in tumor and normal tissue, N0 group and N1group by RT-qPCR.

We further evaluated the performance of the three-gene model for Cuprotosis. First, the results of univariate Cox regression analysis showed that BUB1 (HR: 3.576, 95% CI: 2.14063-5.97377, *P*<0.001), FDX1 (HR: 0.4046, 95% CI: 0.22222-0.73668, *P*= 0.00308), RPL3 (HR: 0.5724, 95% CI: 0.34249-0.95663, *P*=0.03324), T stage (HR: 2.2366, 95% CI: 1.31427-3.80621, *P*=0.003) and N stage (HR: 2.65774, 95% CI: 1.13725-6.21111, *P*=0.02401) were significantly associated with thyroid cancer prognosis (**Figure 5A**). According to multivariate Cox regression analysis, BUB1 was an independent risk factor for thyroid cancer (HR: 4.21965, 95% CI: 2.10531-8.45738, *P*=0.00005; **Figure 5B**). This means that BUB1 could be an independent prognostic factor for thyroid cancer.

In addition, a nomogram model for THCA was constructed based on the clinical features and the three selected CRGs. **Figure 5C** shows the ability to predict the 1-year, 3-year and 5-year DFS probability of THCA patients. Most importantly, by combining the T stage and BUB1, the calibration curves confirmed that the 1-, 3- and 5-year survival probability predicted by the nomogram (red, yellow, grey) is close to the actual survival (**Figure 5D**). To validate the expression in practice, twenty paired tissue samples were collected from clinical patients at our center. As shown in **Figure 5E**, BUB1 was differentially expressed in tumor tissue compared to normal thyroid tissue. This was consistent with the TCGA database. In addition, we compared the expression of BUB1 in the N0 group (without lymph node metastasis) and the N1 group (with lymph node metastasis), unfortunately, there is no significant difference between the two groups. This may be due to the small number of samples. The above analyses suggest that BUB1, FDX1 and RPL3 may be associated with THCA survival.

### 3.5 The three CRGs were involved in immune infiltration

**Figure 6:**
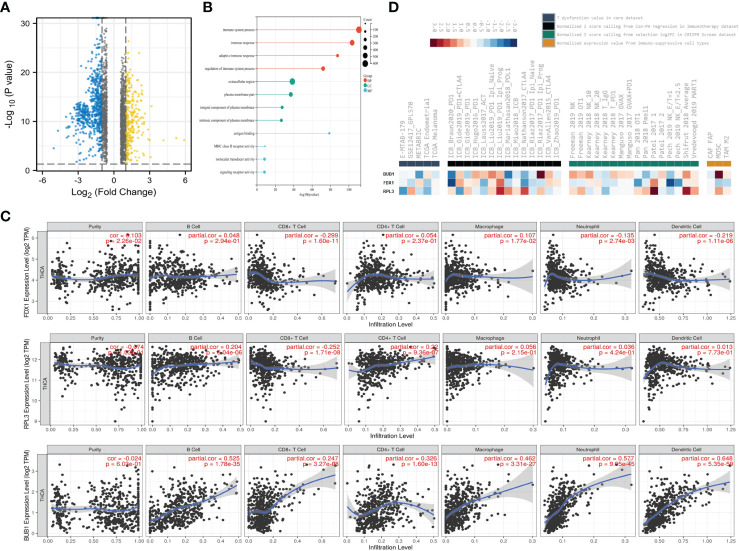
The three CRGs were involved in Immune Infiltration. **(A)** Volcano plots showing DEGs (Fold change <1.5); **(B)** GO enrichment analysis of DEGs; **(C)** Correlation between FDX1, RPL3, BUB1 expression and immune infiltration in THCA in the TIMER database; **(D)** Rank(ascendingly/descendingly) of FDX1, RPL3, BUB1 based on the average score of a group with multiple cohorts.

To further investigate the possible mechanisms of CRGs in THCA, we first divided patients into a low-risk group and a high-risk group based on the risk score. As shown in the volcano curve (**Figure 6A**), 624 DEGs in the high-risk groups were significantly upregulated, while 267 DEGs were downregulated. Interestingly, further analysis at GO indicated that the biological process of these DEGs was mainly related to the immune system process, immune response and adaptive immune response (**Figure 6B**).

Therefore, immune infiltration analysis was applied to investigate their possible roles and relationships (**Figure 6C**). The expression level of FDX1 was positively associated with the immune infiltration level of macrophages (*P*=1.77×10^-2^), but negatively correlated with CD8+T cells (*P*=1.6×10^-11^), neutrophils (*P*=2.74×10^-3^) and dendritic cells (*P*=1.11×10^-6^). However, RPL3 was positively correlated with B cells (*P*= 6.04×10^-6^), CD4+ cells (*P* =9.36×10^-7^) and negatively correlated with CD8+T cells (*P* =1.71×10^-8^). In addition, BUB1 was positively associated with the frequency of B cells (*P* =1.78×10^-35^), CD8+T cells (*P*=3.27×10^-8^), CD4+ cells (*P* =1.6×10^-13^), macrophages (*P* =3.31×10^-27^), neutrophils (*P*=9.95×10^-45^) and dendritic cells (*P*=5.35×10^-59^).

Finally, the database OASIS (13) was annotated to investigate the potential therapeutic targets in synergy with immune checkpoint blockade (ICB) (**Figure 6D**). Interestingly, BUB1 was among the top targets of this module, rendering the tumor microenvironment resistant to ICB. High RPL3 expression was associated with T cell dysfunction phenotypes in TCGA endometrial datasets (**Figure 6D** left panel). Low expression of BUB1 was also associated with poorer ICB outcomes in renal and bladder cancers and in treatment-naïve melanomas treated with ICB (**Figure 6D**, second panel from left). Among the cell types promoting T cell exclusion, myeloid suppressor cells had very high levels of BUB1 expression (**Figure 6D**, right). This module prioritizes BUB1 with the best potential for developing combination immunotherapies. These data suggest that the three CRGs may be involved in regulating immune infiltration.

### 3.6 ATM pathway involved in the regulation of cell death in THCA

**Figure 7:**
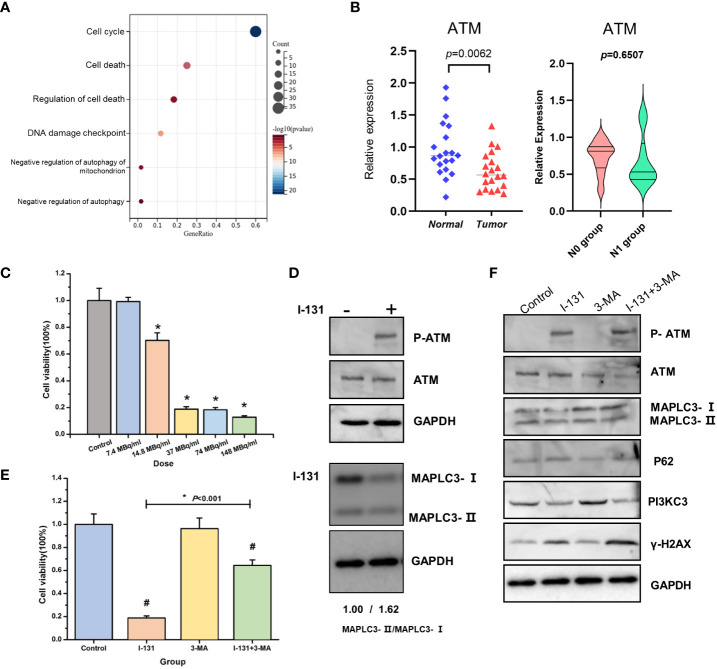
ATM pathway involved in the regulation of cell death in THCA. **(A)** KEGG enrichment analysis of BUB1, FDX1, RPL3 related genes set; **(B)** The expression of ATM in thyroid cancer tissue and normal tissue, N0 group and N1 group; **(C)** Cell viability was estimated in the different doses of I-131; **(D)** The effect of I-131 in ATM, P-ATM and MAPLC3-I/MAPLC3-II; **(E)** Cell viability of control, I-131, 3-MA, I-131 + 3-MA group; **(F)** The expression of P-ATM, ATM, MAPLC3-I/MAPLC3-II, P62, PI3KC3, γ-H2AX, GAPDH of control, I-131, 3-MA, I-131 + 3-MA group. *p < 0.05; #p <0.05 (control vs I-131; 3-MA vs I-131+3-MA).

Our previous work had confirmed that the ATM pathway mainly regulates autophagy induced by irradiation. However, I-131 treatment is the usual therapy, especially for complex and aggressive thyroid cancer. Excitingly, ATM was enriched based on a serious enrichment analysis and was present in the relative functions and pathways of CRGs (**Figure 7A**). This aroused our desire to further investigate the possible role of ATM in regulating cell death in THCA. First, we validated the differential expression of ATM in the paired tissue samples. The expression of ATM in tumor tissue is lower than in normal tissue (*P*=0.0062). in addition, we compared the expression of ATM between the N0 group and N1 group, the median of ATM expression in N1group is lower than N0 group, but there is no significant difference (**Figure 7B**). Then, we investigated its functional role in the thyroid cancer cell line TPC-1. As shown in **Figure 7C**, cell viability was inhibited by increasing I-131 dose in a dose-dependent manner. Considering the minimum effective dose, we chose 14.8 MBq/ml as the treatment dose in the following experiment. According to the Western blot in **Figure 7D**, phosphorylation of ATM was significantly increased after I-131 treatment, implying that the ATM signaling pathway could be activated by I-131 treatment in thyroid cancer. However, as a classic marker of autophagy, an increase in MAPLC3- II/MAPLC3-I was also detected (1.62 vs. 1.00). The common autophagy inhibitor (3- MA) was selected to further investigate the specific role of autophagy in I-131-induced cell death. As shown in **Figure 7E**, only treatment with I-131 could significantly inhibit cell viability, but this could be reversed by combined pre-treatment with 3- MA. Further Western blot showed that ATM is indeed involved in the regulation of autophagy. As shown in **Figure 7F**, I-131 induced an obvious occurrence of phosphorylation of ATM, as well as the increase of MAPLC3-II/MAPLC3-I, and the higher expression of γ-H2AX. However, when pre-treated with 3MA together with I-131, both the phosphorylation of ATM and expression of γ-H2AX was further elevated, however, the MAPLC3-II/MAPLC3-I were inhibited. That indicated that ATM might play a potential role in the I-131 induced autophagy and DNA damage. In addition, gama-H2AX, the DNA damage response marker, was also found to be affected. This suggests that autophagy may be involved in I-131-induced cell death. As there were no standard methods and markers to directly indicate Cuprotosis levels. Whether the ATM pathway specifically regulates Cuprotosis needs further validation in the future.

## 4 Discussion

Cuprotosis is a novel cell death mechanism in recent years, a type of copper-dependent cell death that differs from apoptosis, necroptosis and ferroptosis. Our research could provide a new diagnostic model and new insights into the mechanisms of thyroid cancer development and immunotherapy.

Current advances in the field of Cuprotosis are an exciting and growing area of research field (14–19). (**Figure S1A**) Much evidence suggests that CRGs are expressed in several tumors and correlate with the stage of tumor progression and poor prognosis, including renal cell carcinoma, pancreatic cancer, lung adenocarcinoma, bladder cancer, hepatocellular carcinoma and gastric cancer (**Table S1**). Several other studies have found that FDX1 and DLAT are associated with tumor progression. In this study, we compiled the original data and analysis of the dataset published in Tsvetkov’s research. The dataset included not only 10 genes selected by Tsvetkov, but also 148 genes that log FC>1 or <-1. We investigated signature genes for prognosis (OS, DFS, PFS, DSS) in THCA patients based on the TCGA database. Considering this information, we investigated 19 CRGs related to prognosis in THCA. Finally, 16 CRGs were found to be differentially expressed in tumor and normal tissues, which is consistent with the immunohistochemical results. In addition, the 19 genes showed close interaction in the STRING analysis. BRAF, TERT and RAS gene mutations were frequently observed in thyroid cancer. In this study, we found that GPI, QARS, TUT1 and BUB1 were predominant in CRGs. The biological process (BP) of CRGs mainly focuses on cell death. This is considered a logical next step in our ongoing research.

The mortality rate of THCA is lower and largely different from that of other tumors. Metastasis and recurrence in THCA significantly affect prognosis. Life expectancy is an important parameter reflecting the progression of THCA. In this study, we found 11 genes whose expression is indicative of prognosis in DFS. In multivariate analysis, we confirmed that two genes (BUB1 and GINS2) were of prognostic significance. Studies by Ge MQ confirmed that AKR1C1 candidates have differential expression in THCA compared to normal tissue (20).

Overexpression of BUB1 contributes to the morphological progression of clear cell renal carcinoma (21) Shen YL’s research showed that GINS2 can accelerate the growth of glioma cells (22). According to the above results, we analyzed 19 genes with LASSO regression. The three-gene model (FDX1, BUB1, RPL3) was constructed to predict DFS of 1, 3 and 5 years in thyroid cancer (AUC: 0.789, 0.733, 0.757). Some predictive models for thyroid cancer from other studies showed an AUC of OS (0.621, 0.859, 0.842) (20, 23, 24). By combining the T-stage, the calibration curves confirmed that the 1-, 3- and 5-year survival probability predicted by the nomogram (red, yellow, grey) is close to the actual survival. The difference between a Nomogram and LASSO lies in the incorporation of clinical and pathological features. By analyzing the combination of gene expression and clinical pathological features, the predictive role in thyroid cancer prognosis is assessed. After introducing clinical features, it was found that the predictive efficacy of RPL3 and FDX1 for prognosis was inferior to that of BUB1. Therefore, the final model only includes BUB1 as the important gene for predicting prognosis. The nomogram could potentially be a clinically predictive tool for THCA prognosis. Further investigation into the molecular mechanisms of the 3-gene model revealed that immune infiltration plays a role.

There are a large number of bio-informational articles on THCA, most of which focus on overall survival and ferroptosis. In our manuscript, we chose to study DFS, which is more meaningful to patients, based on our clinical experience. Cuprotosis was discovered in March 2022, there are still many doubts about the mechanism of THCA. We tried to investigate some studies on the treatment of Cuprotosis in THCA. In addition, we built a prediction model based on CRGs.

Through immune cell correlation analysis, we discovered a positive correlation between the expression of BUB1, FDX1, RPL3 and the infiltration of most immune cells. Specifically, the expression of BUB1 showed a positive correlation with B cells (p = 1.78×10−35), CD8+T cells (p =3.27×10−8), CD4+ cell (p =1.6×10−13), macrophages (p =3.31× 10−27), neutrophil (p=9.95×10−45). These immune cells, including CD8+ T cells, CD4+ T cells, macrophages, and dendritic cells, play a crucial role in inhibiting tumor growth. The slow progression of most papillary thyroid carcinomas is often associated with a substantial infiltration of immune cells. The specific mechanisms behind this observation require further experimental exploration.

Although we have verified the expression of 19 genes by immunohistochemistry using bio information, we still need to investigate the expression of patients in our area by RT -qPCR and immunohistochemistry. Further studies include the investigation of FDX1, BUB1 and RPL3 in THCA cell lines and the investigation of Cuprotosis signs in THCA. We found that the molecular mechanisms were related to autophagy by analyzing the signaling pathways of 300 genes of the 3-gene model. ATM might be the central gene of the mechanism. So we conducted an independent experiment. We found that I-131 activates the autophagy pathway *via* the ATM pathway. Benkafadar’s research revealed that ATM triggers apoptosis *via* the P53 pathway (25). Therefore, ATM can be used to inhibit cancer cells.

However, there are some limits. First, the data of our study relied mainly on the public database. Therefore, more basic experimental validation might be needed. Furthermore, we need to collect more samples to reduce statistical errors. At the same time, the mechanism of action between Cuproptosis and immune cells needs to be further explored.

**Figure 8:**
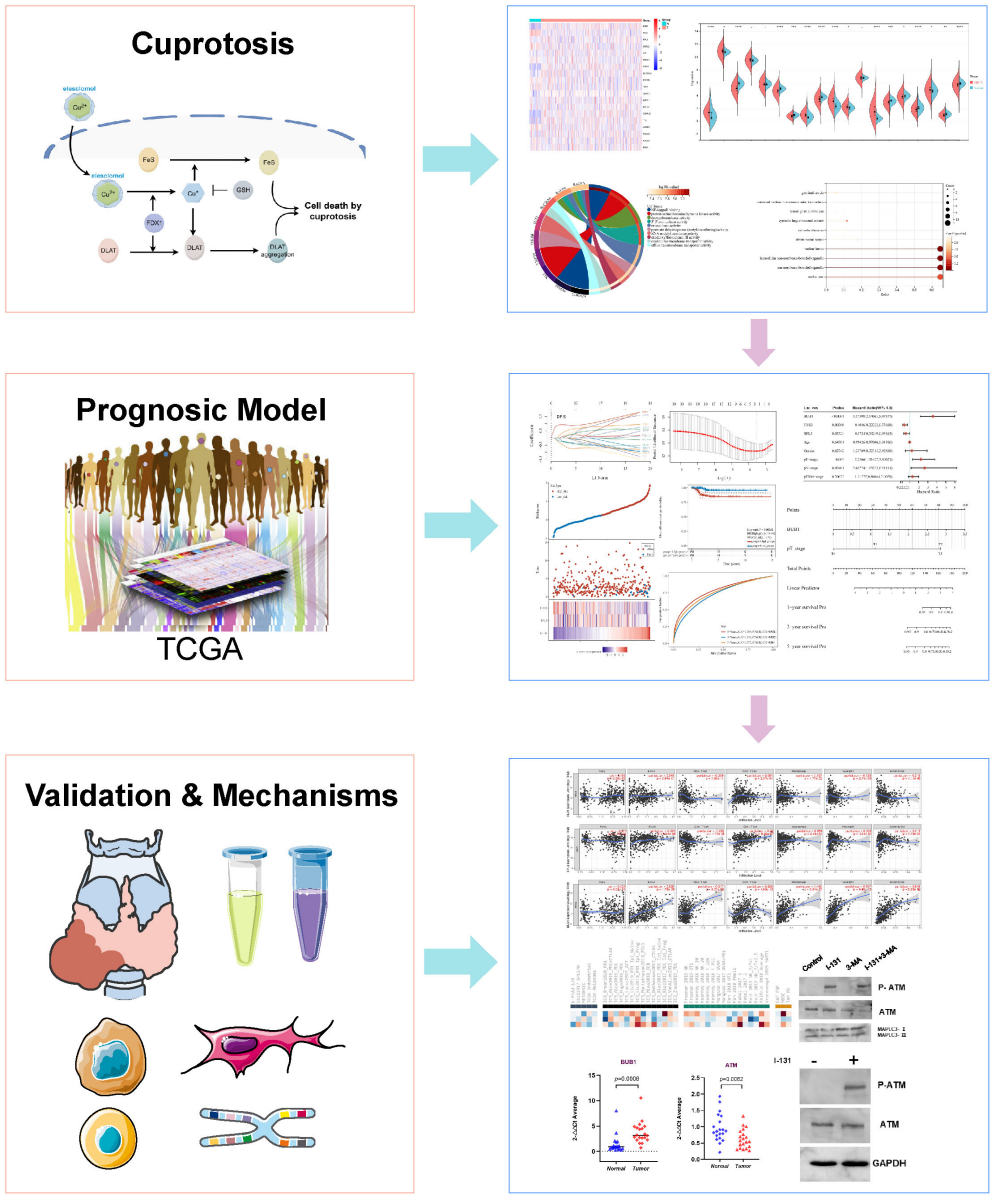
The schematic workflow of the study.

In summary, as shown in **Figure 8**, we found different expressions of CRGs in THCA. In addition, we constructed a novel prognostic model to predict the disease-free survival of THCA. Furthermore, we collected 20 pairs of samples of THCA patients, to verify the expression of CRGs. Additionally, we investigated that CRGs might regulate cell death in THCA through the ATM pathway.

## Conclusion

In conclusion, as shown in **Figure 8**, there are different expressions of CRGs in THCA. We found A novel Prognostic Model to Predict the disease-free survival of THCA. Additionally, we investigated that CRGs might regulate cell death in THCA through the ATM pathway.

## Data availability statement

The raw data supporting the conclusions of this article will be made available by the authors, without undue reservation.

## Ethics statement

The study was conducted in accordance with the Declaration of Helsinki and approved by the China-Japan Union Hospital Institutional Review Board (No.20220804014). Informed consent was obtained from all patients.

## Author contributions

Conceptualization: RD, HS and NL. Data curation: RD, JL, FL and LM. Formal analysis: RD. Funding acquisition: HS and NL. Methodology: RD, JL and FL. Resources: RD. Supervision: GD, HS and NL. Writing – original draft: RD. Writing – review & editing: GD and NL. All authors contributed to the article and approved the submitted version.
