# Supplementary figures and images for: Estimating disease-free survival of thyroid cancer based on novel cuprotosis-related gene model
Source: Front Endocrinol (Lausanne). 2023 Sep 8;14:1209172. doi: 10.3389/fendo.2023.1209172 (PMC10515282; doi:10.3389/fendo.2023.1209172)

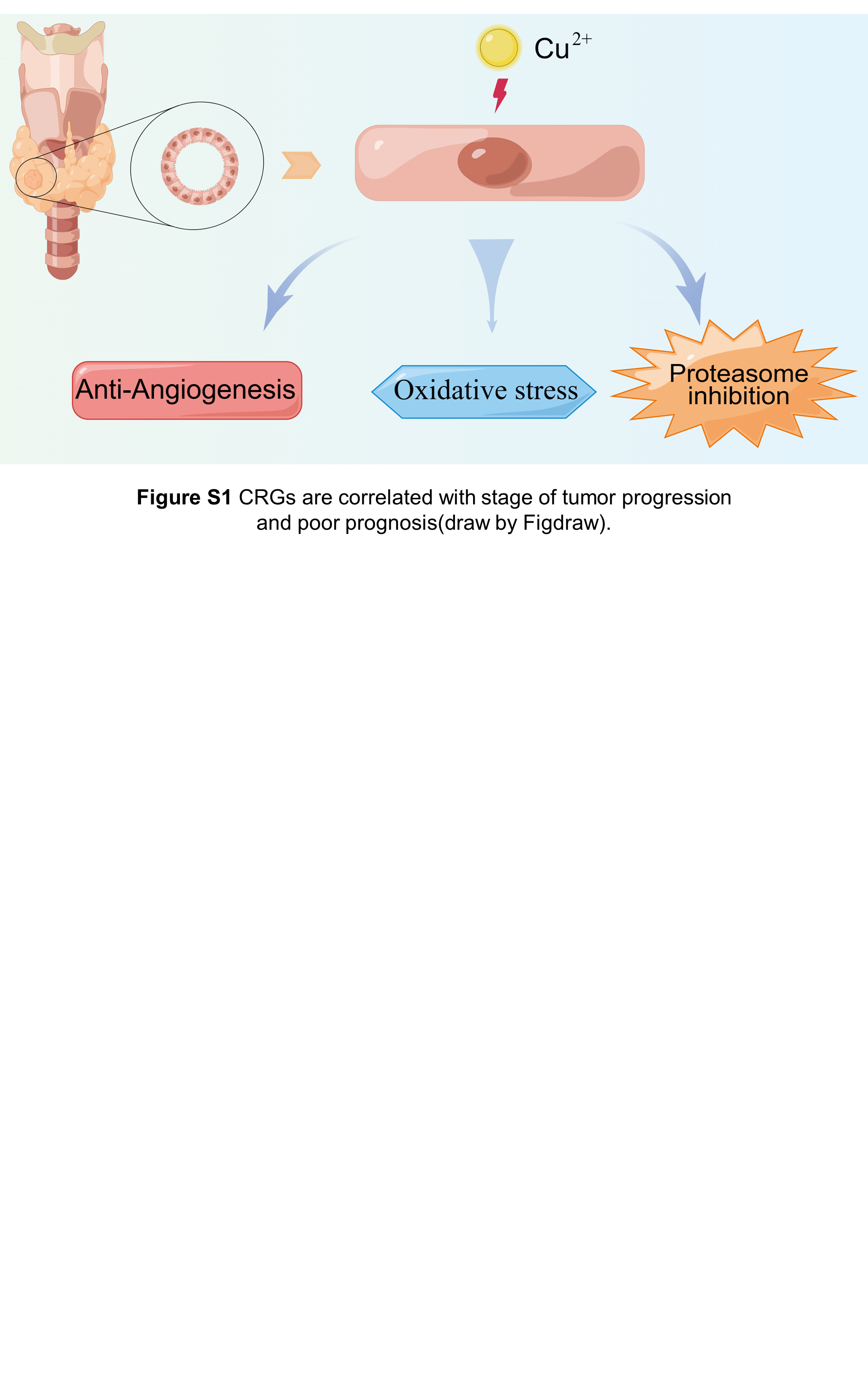

Supplement: Supplementary file 1 [file Image_1.tif]
