# Supplementary material for: Estimating disease-free survival of thyroid cancer based on novel cuprotosis-related gene model
Source: Front Endocrinol (Lausanne). 2023 Sep 8;14:1209172. doi: 10.3389/fendo.2023.1209172 (PMC10515282; doi:10.3389/fendo.2023.1209172)
Supplement: Supplementary file 2 [file Table_1.docx]

**Table S1.** Curoptosis for predicting prognosis in cancer.

| **Author** | **Ref, Year** | **Cancer** |
| --- | --- | --- |
| Liu L et al. | Front Genet. 2023 | Cervical |
| Dong H et al. | Front Surg. 2023 | Gastric |
| Zheng M et al. | J Thorac Dis. 2022 | Lung |
| Jiang A et al. | Cell Biosci. 2022 | Kidney |
| Xu H et al. | World J Clin Cases. 2022 | Esophagus |
| Li W et al. | Front Med. 2022 | Colorectal |
| Xu Y et al. | J Oncol. 2022 | Pancreas |
| Li Z et al. | Front Immunol. 2022 | Breast |
| Song Q et al | Front Immunol. 2022 | Bladder |
| Yang L et al | Front Genet. 2022 | Head & neck |
| Han J et al | J Oncol. 2022 | Soft Tissue Sarcoma |

**Table S2** Clinical characteristics of patients in the Cancer Genome Atlas(TCGA) thyroid cancer patients cohort

| **Clinical**  **Characteristics** | | **Number of Case**  **n=507(%)** |
| --- | --- | --- |
| Age(years) | |  |
|  | Mean (SD) | 47.26(±15.78) |
|  | ＜55 years | 340（67.06%） |
|  | ≥55 years | 167（32.94%） |
| Gender | |  |
|  | Male | 136（26.82%） |
|  | Female | 371（73.18） |
| Stage , n(%) | |  |
|  | Stage I | 285（56.21%） |
|  | Stage II | 52（10.26%） |
|  | Stage III | 113（22.29%） |
|  | Stage IV | 55（10.85%） |
|  | Unknown | 2（0.39%） |
| T status , n (%) | |  |
|  | T1 | 144（28.40%） |
|  | T2 | 167（32.94%） |
|  | T3 | 171（33.73%） |
|  | T4 | 23（4.54%） |
|  | TX | 2（0.39%） |
| N status , n (%) | |  |
|  | N0 | 231（45.56%） |
|  | N1 | 226（44.58%） |
|  | NX | 50（9.86%） |
| M status , n (%) | |  |
|  | MO | 283（55.82%） |
|  | M1 | 9（1.78%） |
|  | MX | 215（42.41%） |
| Overall survival | |  |
|  | living | 491(96.9%) |
|  | Dead | 16(3.1%) |
| Follow time | | 263(135,578) |

**Table S3** Baseline characteristics of papillary thyroid carcinoma (N=20)

| **Case** | **Age** | **Gender** | **Tumor size(mm)** | **N Stage** |
| --- | --- | --- | --- | --- |
| 1 | 33 | Male | 10 | N1a |
| 2 | 49 | Female | 10 | N1a |
| 3 | 27 | Male | 6 | N1b |
| 4 | 24 | Male | 5 | N1b |
| 5 | 27 | Male | 10 | N1a |
| 6 | 34 | Female | 8 | N1a |
| 7 | 33 | Female | 10 | N1a |
| 8 | 33 | Male | 6 | N1a |
| 9 | 32 | Female | 7 | N1a |
| 10 | 34 | Male | 10 | N1a |
| 11 | 44 | Female | 10 | N0 |
| 12 | 52 | Female | 6 | N0 |
| 13 | 53 | Female | 10 | N0 |
| 14 | 48 | Female | 9 | N0 |
| 15 | 41 | Female | 8 | N0 |
| 16 | 43 | Female | 10 | N0 |
| 17 | 44 | Female | 8 | N0 |
| 18 | 55 | Female | 10 | N0 |
| 19 | 57 | Female | 10 | N0 |
| 20 | 34 | Female | 3 | N0 |
